# Retraction: Ménétrier’s Disease and Its Atypical Presentation in Four Siblings

**DOI:** 10.7759/cureus.r78

**Published:** 2023-10-11

**Authors:** Imran H Hassan, Mina Soliman, Ahmad R Shirazi-Nejad

**Affiliations:** 1 Gastroenterology, Barnsley Hospital National Health Service (NHS) Foundation Trust, Barnsley, GBR

This article has been retracted and removed due to a formal request from the family of the patients as formal consent was not obtained from the family prior to submission and publication. The authors and hospital leadership have formally requested this retraction. Due to the privacy concerns of the family, the article contents have been removed as well.

